# (*E*)-4-Chloro-2-[(4-hy­droxy-3-meth­oxy­benzyl­idene)amino]­phenol

**DOI:** 10.1107/S2414314626005523

**Published:** 2026-05-29

**Authors:** Amel Marir, Yamina Boudinar, Ouafa Boukhemis, Amel Djedouani, Erwann Jeanneau, Helen Stoeckli-Evans

**Affiliations:** a Faculté des Sciences Exacte, Université des Fréres Mentouri-Constantine 1, Algeria; b Department of Chemistry, Faculty of Sciences, University of 20 August 1955, Skikda 21000, Algeria; c Laboratory of Physico-Chemistry Research on Surfaces and Interfaces, University of Skikda, 21000, Algeria; d National Biotechnology Research Center (CRBT), Industrial Biotechnology Division, Ali Mendjli New City, UV03 BP E73, Constantine 25000, Algeria; e Unité de Recherche de Chimie de l'Environnement et Moléculaire Structurale Université de Constantine-1 25000 Constantine Algeria; f Ecole Normale Supérieure de Constantine-Assia Djebar, Université Constantine 3, 25000, Ali Mendjli, Algeria; g Laboratoire des Produits Naturels D’Origine Végétale et de Synthése Organique, Faculté des Sciences Exactes, Université des Fréres Mentouri-Constantine 1, Algeria; h Centre de Diffractométrie Henri Longchambon Université Claude Bernard Lyon1 5 rue de la Doua 69100 Villeurbanne France; i Institute of Physics, University of Neuchâtel, Rue Emile-Argand 11, CH-2000 Neuchâtel, Switzerland; University of Aberdeen, United Kingdom

**Keywords:** crystal structure, vanillin, Schiff base, hydrogen bonding

## Abstract

The title compound, C_14_H_12_ClNO_3_ (**I**), was obtained from the reaction of 2-amino-4-chloro phenol with vanillin (4-hy­droxy-3-meth­oxy­benzaldehyde). It crystallizes with two conformationally different mol­ecules in the asymmetric unit. In one of the mol­ecules, the 4-chloro-2(methyl­ene­amino)­phenol moiety is partially positionally disordered with the refined occupancies being 0.850 (2):0.150 (2). In both mol­ecules there are intra­molecular O—H⋯O and O—H⋯N hydrogen bonds, all enclosing *S*(5) ring motifs. The configuration about the azomethine bond is *E* in both mol­ecules. The extended structure features O—H⋯O, C—H⋯Cl, C—H⋯O hydrogen bonds, as well as C—H⋯π and parallel displaced π–π inter­actions, which lead to the formation of a three-dimensional network.

## Abstract 2

The title compound was obtained from the reaction of 2-amino-4-chloro phenol with vanillin (4-hy­droxy-3-meth­oxy­benzaldehyde). It crystallizes with two independent and conformationally different mol­ecules in the asymmetric unit.

## Graphical abstract

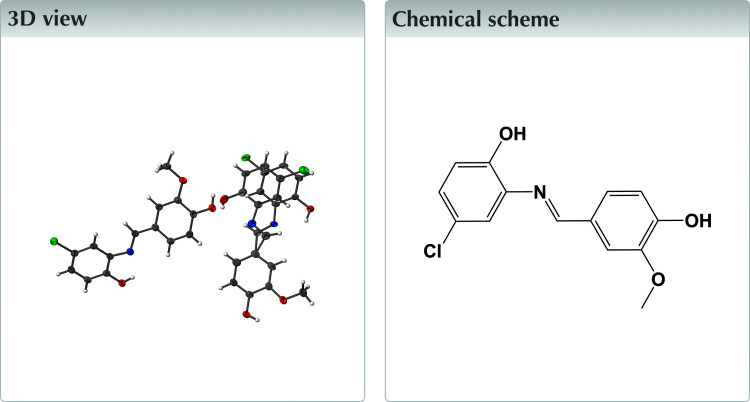

## Structure description

Vanillins have biological importance, for example as a bacterial cofactor involved in the synthesis of folic acid (Robinson, 1966 ▸). Hy­droxy Schiff bases have been widely investigated for their biological, photochromic, and thermochromic properties (Garnovskii *et al.*, 1993 ▸; Hadjoudis *et al.*, 2004 ▸). They therefore represent promising candidates for optical memory and switching devices (Zhao *et al.*, 2007 ▸). The title Schiff base vanillin derivative, C_14_H_12_ClNO_3_ (**I**), was synthesized as part of a broader search for multifunctional imine-bases.

**Figure 1:**
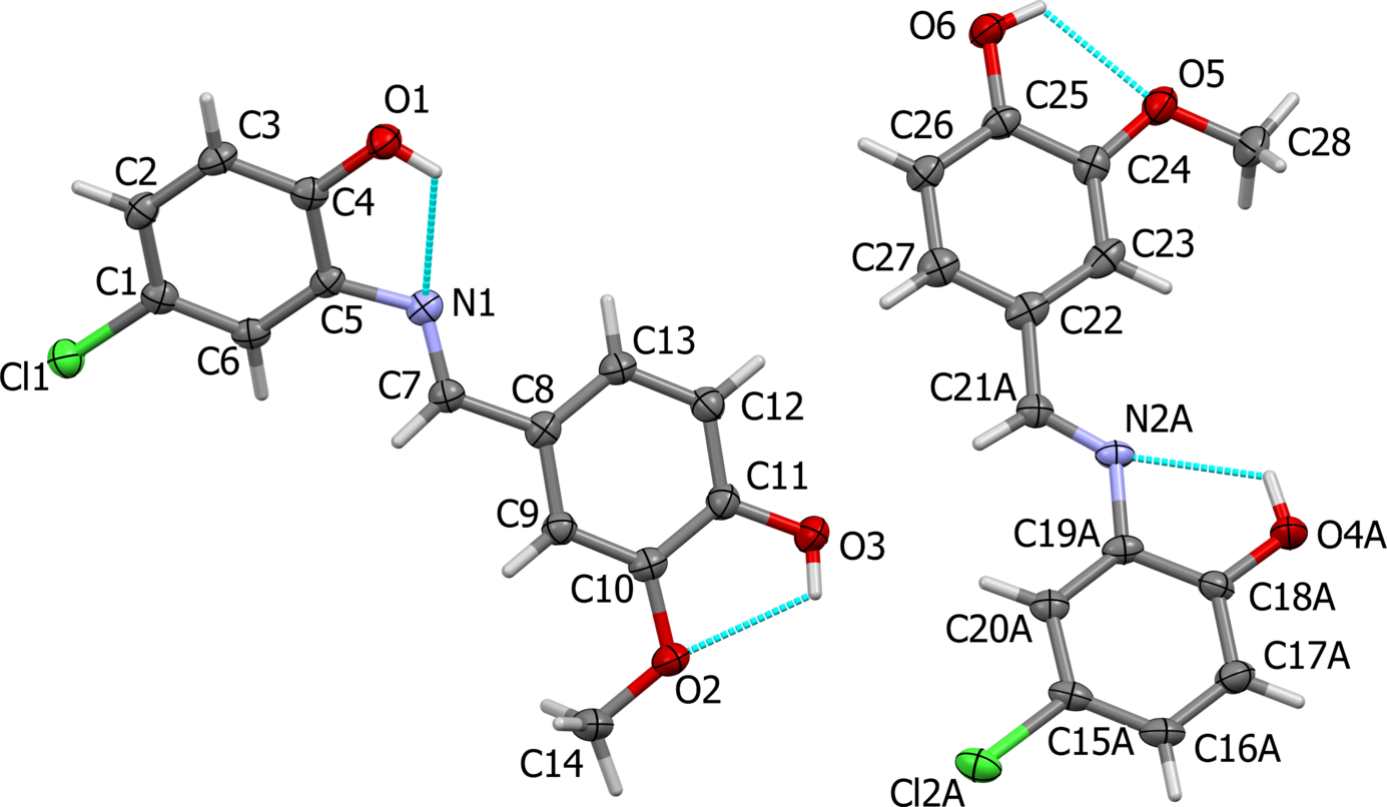
The mol­ecular structure of (**I**) with displacement ellipsoids drawn at the 50% probability level. In this and subsequent figures the various hydrogen bonds (Table 1 ▸) are shown as cyan dashed lines. Only the major disorder component of mol­ecule **2** containing N2*A* is shown.

Compound (**I**) crystallizes with two independent mol­ecules (**1** and **2**) in the asymmetric unit of the monoclinic space group *P*2_1_ with a well defined absolute structure [refined Flack parameter = −0.004 (5)]. The mol­ecular structures of the two mol­ecules are illustrated in Fig. 1 ▸. In both mol­ecules there are intra­molecular O—H⋯O and O—H⋯N hydrogen bonds, all enclosing *S*(5) ring motifs (Fig. 1 ▸ and Table 1 ▸). The configuration about the azomethine bond is *E* in both mol­ecules.

In mol­ecule **2**, the 4-chloro-2(methyl­ene­amino)­phenol moiety is positionally disordered over two sets of sites with refined occupancies (*A*:*B*) of 0.850 (2):0.150 (2). The disorder components are related by a *pseudo* twofold rotation axis on which lies the benzene ring atom C22. In mol­ecule **1** the 2-meth­oxy­phenol ring is inclined to the 4-chloro-2(methyl­ene­amino)­phenol ring by 36.1 (1)°, compared to 5.7 (2)° in mol­ecule **2** for the major component and 7.6 (8)° for the minor component. The disordered 4-chloro-2(methyl­ene­amino)­phenol rings are inclined to each other by 2.7 (8)°. The azomethine bond lengths are normal and of almost the same value: C7=N1, C21*A*=N2*A* and C21*B*=N2*B* are 1.277 (4), 1.279 (4) and 1.269 (12) Å, respectively.

These geometrical parameters are similar to those observed in three similar compounds located in the Cambridge Structural Database (V6.01, last update February 2026; Groom *et al.*, 2016 ▸), namely (*E*)-4-[(4-bromo­phen­yl)imino­meth­yl]-2-meth­oxy­phenol (**II**) (CSD refcode: LEFVID; Fejfarová *et al.*, 2012 ▸), 4-[(4-chloro­phen­yl)imino­meth­yl]-2-meth­oxy­phenol (**III**) (YIFYAO; Shang & Tan, 2007 ▸) and 4-{[(3-chloro-4-fluoro­phen­yl)imino]­meth­yl}-2-meth­oxy­phenol (**IV**) (IREQEF; Suresh Babu *et al.*, 2026 ▸). The dihedral angle between the aromatic rings are 37.9 (1)° for **II**, 44.4 (1)° for **III** and 43.4 (2)° for **IV**, compared to 36.1 (1)° for mol­ecule **1** of the title compound (**I**). The azomethine bond lengths are 1.283 (3) Å for **I**, 1.272 (3) Å for **II** and 1.269 (4) Å for **IV**; close to the values observed in mol­ecules **1** and **2** of compound (**I**).

**Figure 2:**
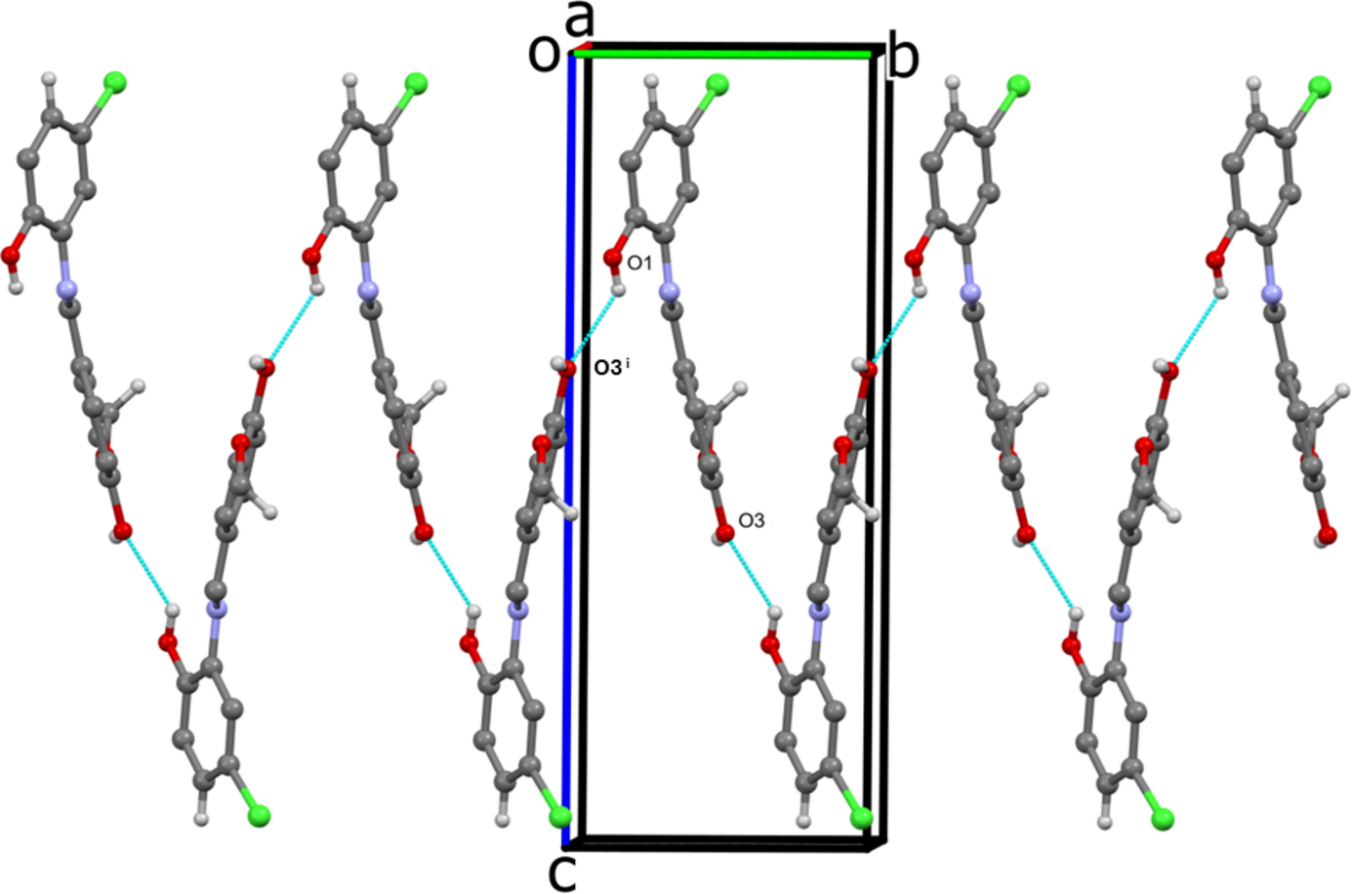
A view along the *a* axis of the helical chain formed by O1—H1⋯O3^i^ hydrogen bonds involving mol­ecules **1** of compound (**I**). Symmetry code: (i) −*x*, *y* − 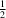, −*z* + 1.

**Figure 3:**
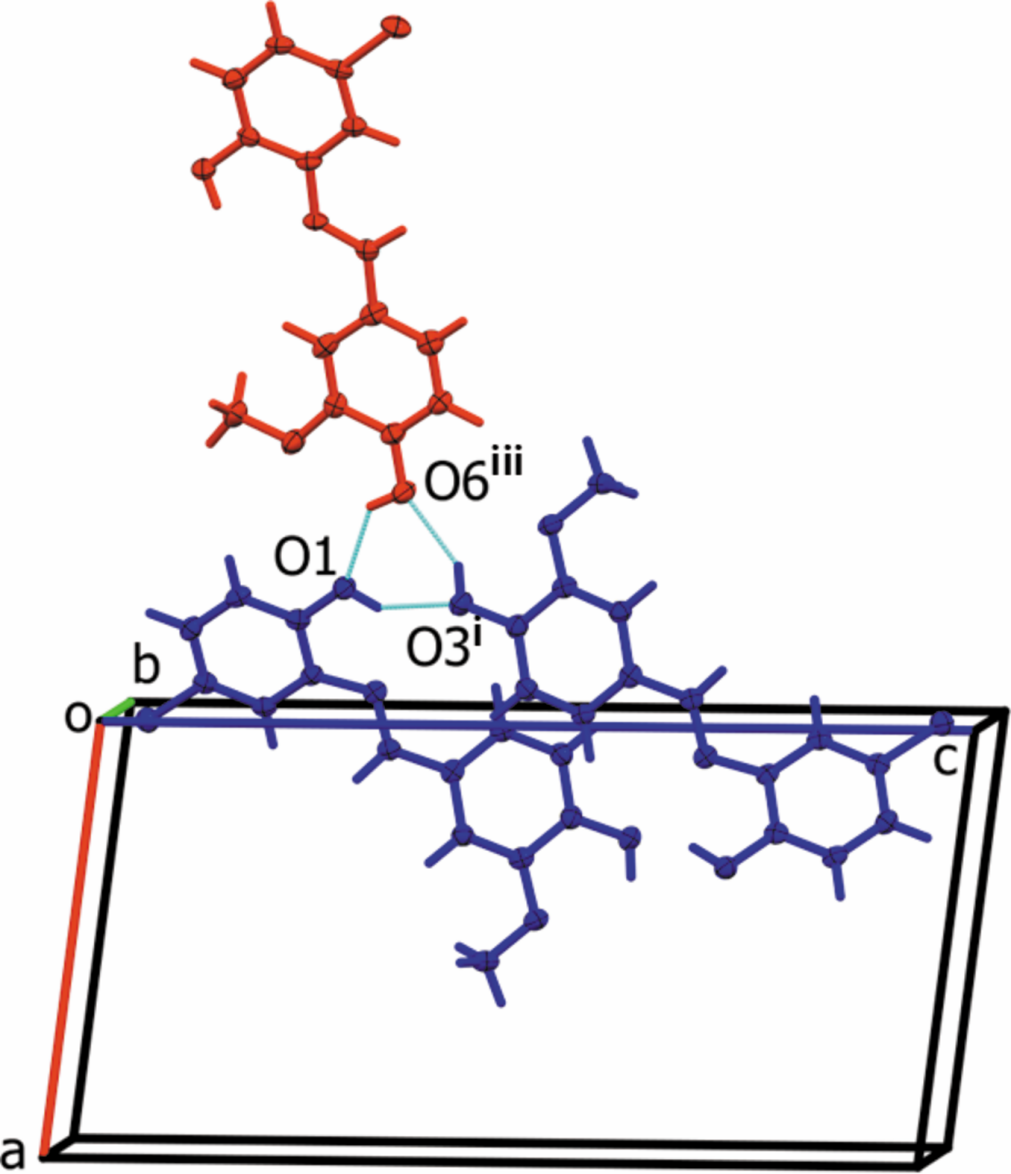
A view along the *b* axis of the hydrogen-bonded 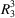
(6) ring motif involving two mol­ecule **1** (blue) and one mol­ecule **2** (red) of compound (**I**). Only the major disorder component of mol­ecule **2** is shown. Symmetry codes: (i) −*x*, *y* − 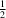, −*z* + 1; (iii) −*x* − 1, *y* + 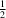, −*z* + 1.

**Figure 4:**
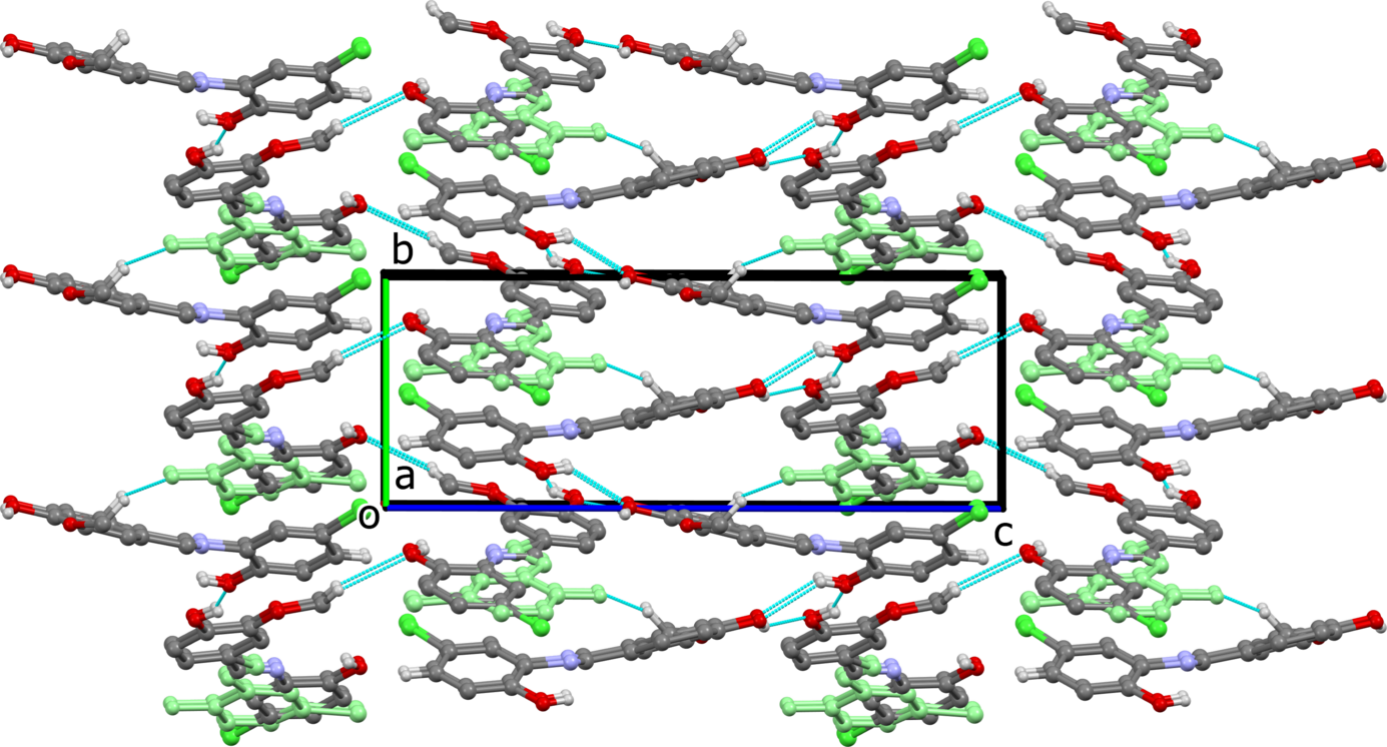
A view along the *a* axis of the packing of compound (**I**). The minor disordered fragment of mol­ecule **2** is shown in pale green.

In the extended structure of (**I**), the **1** mol­ecules are linked by an O1—H1⋯O3^i^ hydrogen bond (Table 1 ▸) forming a helical chain propagating along the *b*-axis direction (Fig. 2 ▸, Table 1 ▸). An inter­esting triangular arrangement with an 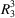
(6) ring motif is formed by O—H⋯O hydrogen bonds involving two **1** mol­ecules and one **2** mol­ecule (Fig. 3 ▸, Table 1 ▸). There are also C—H⋯Cl and C—H⋯O hydrogen bonds present (Table 1 ▸). The combination of all these hydrogen bonds together with C—H⋯π and parallel displaced π–π inter­actions [*Cg*1⋯*Cg*4^i^ = 3.701 (2) Å, where *Cg*1 and *Cg*4 are the centroids of rings C15*A*–C20*A* and C1–C6, respectively] leads to the formation of a three-dimensional network (Fig. 4 ▸).

## Synthesis and crystallization

To a solution of vanillin (0.01 mmol) in ethanol (20 ml) was added a solution of 2-amino-4-chloro phenol (0.01 mmol) also dissolved in ethanol (20 ml). The reaction mixture was stirred for 2.5 h under reflux. The product obtained was filtered off, recrystallized from ethanol solution and then dried *in vacuo* to give compound (**I**) [yield 59%; m.p. 525 K]. Yellow crystals of (**I**), suitable for X-ray analysis, were obtained by slow evaporation of an ethanol solution.

## Refinement

Crystal data, data collection and structure refinement details are summarized in Table 2 ▸. The hydrogen atoms were fixed geometrically (O—H = 0.84 Å, C—H = 0.95–0.98 Å) and allowed to ride on their parent atoms with *U*_iso_(H) = 1.5*U*_eq_(OH and *C*-meth­yl) and 1.2*U*_eq_(C) for other H atoms. The bond lengths and the *U*_iso_/*U*_aniso_ values of the atoms of minor component (*B*) of the disordered moiety of mol­ecule **2** were restrained to be equal to those of the major component (*A*).

## Supplementary Material

Crystal structure: contains datablock(s) global, I. DOI: 10.1107/S2414314626005523/hb4564sup1.cif

Structure factors: contains datablock(s) I. DOI: 10.1107/S2414314626005523/hb4564Isup2.hkl

Supporting information file. DOI: 10.1107/S2414314626005523/hb4564Isup3.cml

CCDC reference: 2448683

Additional supporting information:  crystallographic information; 3D view; checkCIF report

## Figures and Tables

**Table 1:** Hydrogen-bond geometry (Å, °) *Cg*2 is the centroid of the C22–C27 ring.

*D*—H⋯*A*	*D*—H	H⋯*A*	*D*⋯*A*	*D*—H⋯*A*
O1—H1⋯N1	0.84	2.21	2.695 (3)	116
O3—H3⋯O2	0.84	2.25	2.690 (3)	113
O4*A*—H4*A*⋯N2*A*	0.84	2.20	2.675 (4)	116
O6—H6⋯O5	0.84	2.18	2.645 (3)	115
O1—H1⋯O3^i^	0.84	2.26	2.854 (3)	128
O3—H3⋯O6^ii^	0.84	2.02	2.714 (3)	140
O6—H6⋯O1^iii^	0.84	2.05	2.716 (3)	136
C2—H2⋯Cl2*B*^iv^	0.95	2.71	3.449 (8)	135
C14—H14*B*⋯O4*B*^v^	0.98	2.31	2.928 (16)	120
C28—H28*C*⋯O4*A*^vi^	0.98	2.57	3.142 (4)	117
C7—H7⋯*Cg*2^i^	0.95	2.90	3.394 (3)	113

Symmetry codes: (i) 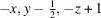; (ii) 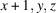; (iii) 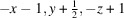; (iv) 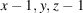; (v) 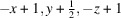; (vi) 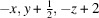.

**Table 2:** Experimental details

Crystal data	
Chemical formula	C_14_H_12_ClNO_3_
*M*_r_	277.70
Crystal system, space group	Monoclinic, *P*2_1_
Temperature (K)	100
*a*, *b*, *c* (Å)	9.5413 (1), 7.0494 (1), 18.9614 (2)
β (°)	97.187 (1)
*V* (Å^3^)	1265.33 (3)
*Z*	4
Radiation type	Cu *K*α
μ (mm^−1^)	2.72
Crystal size (mm)	0.21 × 0.19 × 0.03

Data collection	
Diffractometer	XtaLAB Synergy, Dualflex, HyPix-Arc 100
Absorption correction	Gaussian (*CrysAlis PRO*; Rigaku OD, 2025 ▸)
*T*_min_, *T*_max_	0.583, 1.000
No. of measured, independent and observed [*I* > 2σ(*I*)] reflections	45219, 5122, 4984
*R*_int_	0.032
(sin θ/λ)_max_ (Å^−1^)	0.638

Refinement	
*R*[*F*^2^ > 2σ(*F*^2^)], *wR*(*F*^2^), *S*	0.033, 0.082, 1.07
No. of reflections	5122
No. of parameters	442
No. of restraints	395
H-atom treatment	H-atom parameters constrained
Δρ_max_, Δρ_min_ (e Å^−3^)	0.22, −0.30
Absolute structure	Flack *x* determined using 2147 quotients [(*I*^+^)−(*I*^−^)]/[(*I*^+^)+(*I*^−^)] (Parsons *et al.*, 2013 ▸).
Absolute structure parameter	−0.004 (5)

Computer programs: *CrysAlis PRO* (Rigaku OD, 2025 ▸), *SHELXT2018/2* (Sheldrick, 2015*a* ▸), *OLEX2* (Dolomanov *et al.*, 2009 ▸), *SHELXL 2018/3* (Sheldrick, 2015*b* ▸), *Mercury* (Macrae *et al.*, 2020 ▸), *PLATON* (Spek, 2020 ▸) and *publCIF* (Westrip, 2010 ▸).

## full crystallographic data

### (E)-4-Chloro-2-[(4-hydroxy-3-methoxybenzylidene)amino]phenol Crystal data

**Table:**

C_14_H_12_ClNO_3_	*F*(000) = 576
*M**_r_* = 277.70	*D*_x_ = 1.458 Mg m^−^^3^
Monoclinic, *P*2_1_	Cu *K*α radiation, λ = 1.54184 Å
*a* = 9.5413 (1) Å	Cell parameters from 33083 reflections
*b* = 7.0494 (1) Å	θ = 4.6–77.6°
*c* = 18.9614 (2) Å	µ = 2.72 mm^−^^1^
β = 97.187 (1)°	*T* = 100 K
*V* = 1265.33 (3) Å^3^	Plate, black
*Z* = 4	0.21 × 0.19 × 0.03 mm

### (E)-4-Chloro-2-[(4-hydroxy-3-methoxybenzylidene)amino]phenol Data collection

**Table:**

XtaLAB Synergy, Dualflex, HyPix-Arc 100 diffractometer	5122 independent reflections
Radiation source: micro-focus sealed X-ray tube, PhotonJet (Cu) X-ray Source	4984 reflections with *I* > 2σ(*I*)
Mirror monochromator	*R*_int_ = 0.032
Detector resolution: 10.0000 pixels mm^-1^	θ_max_ = 79.5°, θ_min_ = 4.7°
ω scans	*h* = −11→11
Absorption correction: gaussian (CrysAlisPro; Rigaku OD, 2025)	*k* = −8→8
*T*_min_ = 0.583, *T*_max_ = 1.000	*l* = −24→23
45219 measured reflections	

### (E)-4-Chloro-2-[(4-hydroxy-3-methoxybenzylidene)amino]phenol Refinement

**Table:**

Refinement on *F*^2^	H-atom parameters constrained
Least-squares matrix: full	*w* = 1/[σ^2^(*F*_o_^2^) + (0.0367*P*)^2^ + 0.6939*P*] where *P* = (*F*_o_^2^ + 2*F*_c_^2^)/3
*R*[*F*^2^ > 2σ(*F*^2^)] = 0.033	(Δ/σ)_max_ = 0.001
*wR*(*F*^2^) = 0.082	Δρ_max_ = 0.22 e Å^−^^3^
*S* = 1.07	Δρ_min_ = −0.30 e Å^−^^3^
5122 reflections	Extinction correction: (SHELXL2018/3; Sheldrick, 2015b), Fc^*^=kFc[1+0.001xFc^2^λ^3^/sin(2θ)]^-1/4^
442 parameters	Extinction coefficient: 0.0011 (3)
395 restraints	Absolute structure: Flack *x* determined using 2147 quotients [(*I*^+^)-(*I*^-^)]/[(*I*^+^)+(*I*^-^)] (Parsons *et al.*, 2013).
Primary atom site location: dual	Absolute structure parameter: −0.004 (5)
Hydrogen site location: inferred from neighbouring sites	

### (E)-4-Chloro-2-[(4-hydroxy-3-methoxybenzylidene)amino]phenol Special details

**Table:**

Geometry. All esds (except the esd in the dihedral angle between two l.s. planes) are estimated using the full covariance matrix. The cell esds are taken into account individually in the estimation of esds in distances, angles and torsion angles; correlations between esds in cell parameters are only used when they are defined by crystal symmetry. An approximate (isotropic) treatment of cell esds is used for estimating esds involving l.s. planes.

### (E)-4-Chloro-2-[(4-hydroxy-3-methoxybenzylidene)amino]phenol Fractional atomic coordinates and isotropic or equivalent isotropic displacement parameters (Å^2^)

**Table:**

	*x*	*y*	*z*	*U*_iso_*/*U*_eq_	Occ. (<1)
Cl1	0.01708 (7)	0.48290 (11)	0.03832 (3)	0.03065 (17)	
O1	−0.3043 (2)	0.1573 (3)	0.25322 (10)	0.0280 (4)	
H1	−0.259951	0.174431	0.293848	0.042*	
O2	0.4630 (2)	0.4355 (3)	0.51340 (10)	0.0325 (5)	
O3	0.2779 (2)	0.5063 (4)	0.60610 (10)	0.0355 (5)	
H3	0.364488	0.480950	0.613973	0.053*	
N1	−0.0586 (2)	0.3329 (3)	0.29857 (12)	0.0241 (5)	
C1	−0.0740 (3)	0.3750 (4)	0.10189 (14)	0.0242 (6)	
C2	−0.2020 (3)	0.2866 (4)	0.08055 (15)	0.0259 (6)	
H2	−0.236672	0.276652	0.031502	0.031*	
C3	−0.2792 (3)	0.2123 (4)	0.13181 (16)	0.0262 (6)	
H3A	−0.366968	0.150878	0.117979	0.031*	
C4	−0.2273 (3)	0.2287 (4)	0.20289 (15)	0.0235 (5)	
C5	−0.0967 (3)	0.3146 (4)	0.22449 (14)	0.0218 (5)	
C6	−0.0200 (3)	0.3885 (4)	0.17267 (14)	0.0225 (5)	
H6A	0.068753	0.447708	0.186105	0.027*	
C7	0.0722 (3)	0.3355 (4)	0.32331 (15)	0.0238 (5)	
H7	0.139799	0.311035	0.291697	0.029*	
C8	0.1228 (3)	0.3742 (4)	0.39765 (14)	0.0241 (6)	
C9	0.2686 (3)	0.3771 (4)	0.41894 (14)	0.0243 (6)	
H9	0.331511	0.346927	0.385497	0.029*	
C10	0.3224 (3)	0.4231 (4)	0.48776 (15)	0.0257 (6)	
C11	0.2293 (3)	0.4636 (4)	0.53746 (14)	0.0269 (6)	
C12	0.0850 (3)	0.4613 (4)	0.51677 (14)	0.0266 (6)	
H12	0.022182	0.489326	0.550477	0.032*	
C13	0.0312 (3)	0.4184 (4)	0.44712 (14)	0.0260 (6)	
H13	−0.067986	0.419123	0.433177	0.031*	
C14	0.5597 (3)	0.4389 (5)	0.46143 (16)	0.0362 (7)	
H14A	0.655236	0.464287	0.484917	0.054*	
H14B	0.531856	0.538831	0.426542	0.054*	
H14C	0.558057	0.315888	0.437259	0.054*	
Cl2A	0.61960 (8)	−0.00605 (13)	0.75094 (4)	0.0330 (2)	0.850 (2)
Cl2B	0.5418 (7)	0.0967 (13)	0.9561 (4)	0.0549 (19)	0.150 (2)
O4A	0.2755 (3)	0.3018 (4)	0.95883 (13)	0.0324 (6)	0.850 (2)
H4A	0.192939	0.322718	0.939384	0.049*	0.850 (2)
O4B	0.3188 (16)	0.125 (3)	0.6549 (7)	0.043 (4)	0.150 (2)
H4B	0.268030	0.221346	0.645512	0.065*	0.150 (2)
O5	−0.3574 (2)	0.5535 (3)	0.82344 (10)	0.0322 (5)	
O6	−0.4691 (2)	0.5218 (3)	0.68912 (10)	0.0336 (5)	
H6	−0.503586	0.557855	0.725492	0.050*	
N2A	0.1597 (3)	0.2935 (4)	0.82290 (15)	0.0220 (6)	0.850 (2)
N2B	0.1569 (16)	0.257 (3)	0.7449 (9)	0.029 (3)	0.150 (2)
C15A	0.5165 (4)	0.0862 (6)	0.8128 (2)	0.0263 (8)	0.850 (2)
C15B	0.4796 (17)	0.115 (3)	0.8662 (6)	0.033 (3)	0.150 (2)
C16A	0.5714 (4)	0.0890 (5)	0.88371 (18)	0.0293 (7)	0.850 (2)
H16A	0.663202	0.040906	0.898876	0.035*	0.850 (2)
C16B	0.562 (2)	0.055 (4)	0.8152 (8)	0.031 (3)	0.150 (2)
H16B	0.655735	0.013414	0.829141	0.037*	0.150 (2)
C17A	0.4890 (4)	0.1638 (6)	0.9323 (2)	0.0315 (8)	0.850 (2)
H17A	0.525101	0.168726	0.981242	0.038*	0.850 (2)
C17B	0.5071 (15)	0.056 (3)	0.7440 (8)	0.029 (3)	0.150 (2)
H17B	0.561093	0.008931	0.709024	0.035*	0.150 (2)
C18A	0.3545 (3)	0.2314 (5)	0.90979 (17)	0.0251 (7)	0.850 (2)
C18B	0.3722 (15)	0.127 (3)	0.7246 (7)	0.031 (3)	0.150 (2)
C19A	0.3001 (4)	0.2271 (5)	0.83757 (19)	0.0229 (7)	0.850 (2)
C19B	0.2913 (16)	0.196 (3)	0.7753 (7)	0.029 (3)	0.150 (2)
C20A	0.3837 (3)	0.1539 (5)	0.78864 (18)	0.0243 (6)	0.850 (2)
H20A	0.349394	0.150685	0.739408	0.029*	0.850 (2)
C20B	0.3450 (19)	0.188 (4)	0.8467 (9)	0.029 (3)	0.150 (2)
H20B	0.290374	0.232779	0.881952	0.035*	0.150 (2)
C21A	0.0944 (4)	0.2930 (5)	0.7597 (2)	0.0232 (8)	0.850 (2)
H21A	0.142836	0.251979	0.721620	0.028*	0.850 (2)
C21B	0.0758 (16)	0.308 (4)	0.7896 (9)	0.033 (4)	0.150 (2)
H21B	0.095063	0.314990	0.839954	0.039*	0.150 (2)
C22	−0.0536 (3)	0.3541 (4)	0.74452 (16)	0.0279 (6)	
C23	−0.1313 (3)	0.4268 (4)	0.79672 (15)	0.0290 (6)	
H23	−0.088500	0.437681	0.844539	0.035*	
C24	−0.2693 (3)	0.4822 (4)	0.77865 (14)	0.0256 (5)	
C25	−0.3328 (3)	0.4665 (4)	0.70778 (14)	0.0264 (6)	
C26	−0.2570 (3)	0.3920 (4)	0.65653 (15)	0.0269 (6)	
H26	−0.300329	0.378857	0.608869	0.032*	
C27	−0.1180 (3)	0.3367 (4)	0.67500 (16)	0.0290 (6)	
H27	−0.066043	0.286383	0.639691	0.035*	
C28	−0.2971 (4)	0.5866 (5)	0.89510 (16)	0.0376 (7)	
H28A	−0.214351	0.669073	0.895543	0.056*	
H28B	−0.268475	0.465409	0.917827	0.056*	
H28C	−0.367156	0.647938	0.921164	0.056*	

### (E)-4-Chloro-2-[(4-hydroxy-3-methoxybenzylidene)amino]phenol Atomic displacement parameters (Å^2^)

**Table:**

	*U*^11^	*U*^22^	*U*^33^	*U*^12^	*U*^13^	*U*^23^
Cl1	0.0304 (3)	0.0323 (3)	0.0295 (3)	−0.0008 (3)	0.0046 (2)	0.0043 (3)
O1	0.0267 (10)	0.0309 (11)	0.0258 (10)	−0.0040 (8)	0.0005 (8)	0.0024 (8)
O2	0.0218 (10)	0.0450 (13)	0.0295 (10)	0.0000 (9)	−0.0017 (7)	−0.0051 (9)
O3	0.0277 (10)	0.0526 (14)	0.0253 (9)	0.0005 (11)	−0.0008 (7)	−0.0089 (10)
N1	0.0256 (12)	0.0198 (11)	0.0262 (11)	0.0001 (9)	0.0008 (9)	−0.0008 (9)
C1	0.0247 (14)	0.0195 (13)	0.0285 (13)	0.0030 (11)	0.0037 (10)	0.0012 (10)
C2	0.0286 (15)	0.0231 (14)	0.0249 (13)	0.0016 (11)	−0.0015 (11)	0.0002 (11)
C3	0.0230 (14)	0.0239 (14)	0.0303 (14)	−0.0021 (11)	−0.0028 (11)	0.0008 (11)
C4	0.0229 (13)	0.0186 (12)	0.0288 (14)	0.0026 (10)	0.0029 (11)	0.0030 (10)
C5	0.0221 (13)	0.0187 (13)	0.0236 (13)	0.0030 (10)	−0.0005 (10)	−0.0014 (10)
C6	0.0199 (12)	0.0171 (13)	0.0300 (13)	0.0029 (10)	0.0015 (10)	−0.0014 (10)
C7	0.0240 (14)	0.0211 (13)	0.0262 (13)	0.0013 (11)	0.0025 (10)	−0.0002 (10)
C8	0.0276 (14)	0.0190 (13)	0.0252 (13)	−0.0001 (11)	0.0009 (10)	0.0007 (10)
C9	0.0229 (13)	0.0246 (14)	0.0251 (13)	0.0012 (10)	0.0023 (10)	−0.0005 (10)
C10	0.0235 (13)	0.0231 (13)	0.0295 (13)	−0.0004 (11)	0.0001 (10)	0.0008 (11)
C11	0.0281 (13)	0.0289 (16)	0.0231 (12)	−0.0007 (12)	0.0008 (10)	−0.0020 (11)
C12	0.0266 (13)	0.0271 (15)	0.0266 (13)	−0.0014 (12)	0.0055 (10)	−0.0011 (11)
C13	0.0221 (13)	0.0274 (13)	0.0280 (13)	−0.0011 (11)	0.0007 (10)	−0.0001 (11)
C14	0.0208 (14)	0.050 (2)	0.0369 (15)	0.0010 (13)	0.0024 (11)	−0.0028 (14)
Cl2A	0.0254 (4)	0.0310 (4)	0.0442 (5)	0.0045 (4)	0.0099 (3)	−0.0034 (4)
Cl2B	0.035 (3)	0.075 (5)	0.052 (4)	−0.007 (3)	−0.006 (3)	−0.008 (3)
O4A	0.0232 (12)	0.0463 (15)	0.0276 (12)	0.0092 (11)	0.0026 (9)	0.0019 (11)
O4B	0.042 (9)	0.052 (10)	0.038 (8)	0.015 (7)	0.014 (6)	−0.002 (7)
O5	0.0323 (11)	0.0374 (12)	0.0253 (10)	−0.0003 (9)	−0.0035 (8)	−0.0036 (9)
O6	0.0261 (10)	0.0454 (14)	0.0276 (10)	0.0077 (9)	−0.0030 (8)	−0.0076 (9)
N2A	0.0168 (13)	0.0181 (12)	0.0306 (14)	0.0019 (10)	0.0016 (11)	0.0018 (10)
N2B	0.028 (6)	0.026 (6)	0.035 (6)	−0.007 (6)	0.006 (6)	0.002 (5)
C15A	0.018 (2)	0.0203 (18)	0.0416 (19)	0.0005 (15)	0.0085 (14)	0.0003 (14)
C15B	0.027 (5)	0.032 (5)	0.039 (5)	−0.005 (4)	0.003 (4)	0.001 (4)
C16A	0.0170 (16)	0.0282 (17)	0.0425 (18)	0.0013 (13)	0.0022 (13)	0.0041 (15)
C16B	0.020 (6)	0.026 (6)	0.046 (6)	−0.010 (6)	0.005 (6)	0.000 (5)
C17A	0.0223 (19)	0.038 (2)	0.0330 (19)	0.0007 (16)	−0.0021 (15)	0.0047 (16)
C17B	0.021 (6)	0.028 (6)	0.041 (6)	0.001 (5)	0.010 (5)	0.003 (6)
C18A	0.0191 (15)	0.0275 (16)	0.0295 (17)	0.0022 (13)	0.0063 (12)	0.0043 (13)
C18B	0.033 (7)	0.025 (6)	0.037 (7)	−0.003 (6)	0.008 (6)	0.001 (6)
C19A	0.0177 (18)	0.0164 (17)	0.0339 (17)	0.0006 (13)	0.0004 (14)	0.0033 (13)
C19B	0.026 (5)	0.027 (5)	0.033 (5)	−0.005 (5)	0.005 (5)	0.001 (5)
C20A	0.0224 (16)	0.0196 (15)	0.0309 (16)	−0.0016 (13)	0.0028 (12)	0.0001 (13)
C20B	0.022 (5)	0.025 (5)	0.039 (5)	−0.003 (5)	0.001 (5)	0.003 (5)
C21A	0.0232 (19)	0.0192 (16)	0.0275 (19)	−0.0009 (14)	0.0034 (15)	0.0023 (16)
C21B	0.031 (7)	0.030 (7)	0.038 (8)	−0.008 (6)	0.006 (7)	0.000 (7)
C22	0.0283 (15)	0.0166 (13)	0.0368 (15)	−0.0014 (11)	−0.0035 (11)	0.0040 (11)
C23	0.0329 (15)	0.0193 (13)	0.0315 (14)	−0.0044 (11)	−0.0087 (11)	0.0029 (11)
C24	0.0317 (13)	0.0186 (11)	0.0261 (12)	−0.0008 (12)	0.0016 (10)	−0.0001 (11)
C25	0.0247 (13)	0.0233 (14)	0.0299 (13)	0.0012 (12)	−0.0022 (10)	0.0022 (12)
C26	0.0286 (14)	0.0248 (14)	0.0260 (13)	0.0016 (12)	−0.0021 (10)	−0.0019 (11)
C27	0.0302 (15)	0.0216 (14)	0.0346 (15)	0.0004 (12)	0.0010 (12)	0.0011 (11)
C28	0.0357 (17)	0.049 (2)	0.0267 (15)	−0.0060 (15)	−0.0014 (12)	−0.0084 (14)

### (E)-4-Chloro-2-[(4-hydroxy-3-methoxybenzylidene)amino]phenol Geometric parameters (Å, º)

**Table:**

Cl1—C1	1.746 (3)	O6—H6	0.8400
O1—H1	0.8400	O6—C25	1.361 (3)
O1—C4	1.371 (3)	N2A—C19A	1.414 (4)
O2—C10	1.371 (3)	N2A—C21A	1.279 (4)
O2—C14	1.432 (4)	N2B—C19B	1.407 (13)
O3—H3	0.8400	N2B—C21B	1.269 (12)
O3—C11	1.360 (3)	C15A—C16A	1.381 (5)
N1—C5	1.412 (3)	C15A—C20A	1.378 (4)
N1—C7	1.277 (4)	C15B—C16B	1.383 (10)
C1—C2	1.385 (4)	C15B—C20B	1.390 (10)
C1—C6	1.379 (4)	C16A—H16A	0.9500
C2—H2	0.9500	C16A—C17A	1.387 (5)
C2—C3	1.393 (4)	C16B—H16B	0.9500
C3—H3A	0.9500	C16B—C17B	1.386 (10)
C3—C4	1.381 (4)	C17A—H17A	0.9500
C4—C5	1.400 (4)	C17A—C18A	1.385 (5)
C5—C6	1.397 (4)	C17B—H17B	0.9500
C6—H6A	0.9500	C17B—C18B	1.387 (9)
C7—H7	0.9500	C18A—C19A	1.403 (4)
C7—C8	1.457 (4)	C18B—C19B	1.392 (9)
C8—C9	1.400 (4)	C19A—C20A	1.395 (4)
C8—C13	1.395 (4)	C19B—C20B	1.387 (9)
C9—H9	0.9500	C20A—H20A	0.9500
C9—C10	1.380 (4)	C20B—H20B	0.9500
C10—C11	1.403 (4)	C21A—H21A	0.9500
C11—C12	1.384 (4)	C21A—C22	1.470 (5)
C12—H12	0.9500	C21B—H21B	0.9500
C12—C13	1.389 (4)	C21B—C22	1.447 (13)
C13—H13	0.9500	C22—C23	1.406 (4)
C14—H14A	0.9800	C22—C27	1.388 (4)
C14—H14B	0.9800	C23—H23	0.9500
C14—H14C	0.9800	C23—C24	1.375 (4)
Cl2A—C15A	1.747 (4)	C24—C25	1.407 (3)
Cl2B—C15B	1.739 (11)	C25—C26	1.385 (4)
O4A—H4A	0.8400	C26—H26	0.9500
O4A—C18A	1.362 (4)	C26—C27	1.385 (4)
O4B—H4B	0.8400	C27—H27	0.9500
O4B—C18B	1.356 (12)	C28—H28A	0.9800
O5—C24	1.363 (3)	C28—H28B	0.9800
O5—C28	1.427 (3)	C28—H28C	0.9800
			
C4—O1—H1	109.5	C15A—C16A—C17A	118.3 (3)
C10—O2—C14	116.3 (2)	C17A—C16A—H16A	120.8
C11—O3—H3	109.5	C15B—C16B—H16B	119.9
C7—N1—C5	119.0 (2)	C15B—C16B—C17B	120.1 (16)
C2—C1—Cl1	119.4 (2)	C17B—C16B—H16B	119.9
C6—C1—Cl1	119.0 (2)	C16A—C17A—H17A	119.8
C6—C1—C2	121.6 (3)	C18A—C17A—C16A	120.4 (4)
C1—C2—H2	120.4	C18A—C17A—H17A	119.8
C1—C2—C3	119.3 (3)	C16B—C17B—H17B	120.5
C3—C2—H2	120.4	C16B—C17B—C18B	118.9 (15)
C2—C3—H3A	120.2	C18B—C17B—H17B	120.5
C4—C3—C2	119.5 (3)	O4A—C18A—C17A	119.1 (3)
C4—C3—H3A	120.2	O4A—C18A—C19A	120.4 (3)
O1—C4—C3	119.4 (2)	C17A—C18A—C19A	120.5 (3)
O1—C4—C5	119.4 (2)	O4B—C18B—C17B	118.5 (13)
C3—C4—C5	121.3 (3)	O4B—C18B—C19B	120.3 (12)
C4—C5—N1	116.1 (2)	C17B—C18B—C19B	121.2 (13)
C6—C5—N1	125.0 (2)	C18A—C19A—N2A	114.2 (3)
C6—C5—C4	118.7 (2)	C20A—C19A—N2A	126.7 (3)
C1—C6—C5	119.6 (2)	C20A—C19A—C18A	119.0 (3)
C1—C6—H6A	120.2	C18B—C19B—N2B	112.3 (13)
C5—C6—H6A	120.2	C20B—C19B—N2B	128.1 (13)
N1—C7—H7	118.4	C20B—C19B—C18B	119.5 (14)
N1—C7—C8	123.3 (3)	C15A—C20A—C19A	119.0 (3)
C8—C7—H7	118.4	C15A—C20A—H20A	120.5
C9—C8—C7	118.6 (2)	C19A—C20A—H20A	120.5
C13—C8—C7	122.2 (3)	C15B—C20B—H20B	120.4
C13—C8—C9	119.1 (2)	C19B—C20B—C15B	119.2 (15)
C8—C9—H9	119.5	C19B—C20B—H20B	120.4
C10—C9—C8	121.0 (3)	N2A—C21A—H21A	119.1
C10—C9—H9	119.5	N2A—C21A—C22	121.7 (4)
O2—C10—C9	125.6 (2)	C22—C21A—H21A	119.1
O2—C10—C11	115.0 (2)	N2B—C21B—H21B	128.8
C9—C10—C11	119.4 (2)	N2B—C21B—C22	102.4 (13)
O3—C11—C10	121.3 (2)	C22—C21B—H21B	128.8
O3—C11—C12	118.8 (2)	C23—C22—C21A	123.2 (3)
C12—C11—C10	119.9 (2)	C23—C22—C21B	98.7 (8)
C11—C12—H12	119.7	C27—C22—C21A	117.4 (3)
C11—C12—C13	120.6 (2)	C27—C22—C21B	141.7 (8)
C13—C12—H12	119.7	C27—C22—C23	119.4 (3)
C8—C13—H13	120.0	C22—C23—H23	120.0
C12—C13—C8	120.0 (3)	C24—C23—C22	120.1 (2)
C12—C13—H13	120.0	C24—C23—H23	120.0
O2—C14—H14A	109.5	O5—C24—C23	126.5 (2)
O2—C14—H14B	109.5	O5—C24—C25	113.5 (2)
O2—C14—H14C	109.5	C23—C24—C25	120.0 (3)
H14A—C14—H14B	109.5	O6—C25—C24	120.6 (2)
H14A—C14—H14C	109.5	O6—C25—C26	119.4 (2)
H14B—C14—H14C	109.5	C26—C25—C24	120.0 (2)
C18A—O4A—H4A	109.5	C25—C26—H26	120.1
C18B—O4B—H4B	109.5	C25—C26—C27	119.8 (3)
C24—O5—C28	116.6 (2)	C27—C26—H26	120.1
C25—O6—H6	109.5	C22—C27—H27	119.6
C21A—N2A—C19A	121.5 (3)	C26—C27—C22	120.7 (3)
C21B—N2B—C19B	114.5 (14)	C26—C27—H27	119.6
C16A—C15A—Cl2A	118.8 (3)	O5—C28—H28A	109.5
C20A—C15A—Cl2A	118.6 (3)	O5—C28—H28B	109.5
C20A—C15A—C16A	122.6 (3)	O5—C28—H28C	109.5
C16B—C15B—Cl2B	120.5 (11)	H28A—C28—H28B	109.5
C16B—C15B—C20B	120.9 (13)	H28A—C28—H28C	109.5
C20B—C15B—Cl2B	118.6 (11)	H28B—C28—H28C	109.5
C15A—C16A—H16A	120.8		
			
Cl1—C1—C2—C3	−175.5 (2)	O6—C25—C26—C27	179.5 (3)
Cl1—C1—C6—C5	175.5 (2)	N2A—C19A—C20A—C15A	−176.7 (4)
O1—C4—C5—N1	−4.4 (4)	N2A—C21A—C22—C23	3.6 (5)
O1—C4—C5—C6	−179.3 (2)	N2A—C21A—C22—C27	−175.9 (3)
O2—C10—C11—O3	−1.8 (4)	N2B—C19B—C20B—C15B	178 (2)
O2—C10—C11—C12	178.0 (3)	N2B—C21B—C22—C23	−174.2 (16)
O3—C11—C12—C13	180.0 (3)	N2B—C21B—C22—C27	11 (3)
N1—C5—C6—C1	−174.6 (2)	C15A—C16A—C17A—C18A	0.8 (6)
N1—C7—C8—C9	−178.8 (3)	C15B—C16B—C17B—C18B	3 (4)
N1—C7—C8—C13	−2.4 (4)	C16A—C15A—C20A—C19A	−0.5 (6)
C1—C2—C3—C4	0.2 (4)	C16A—C17A—C18A—O4A	179.2 (3)
C2—C1—C6—C5	−1.2 (4)	C16A—C17A—C18A—C19A	−0.7 (6)
C2—C3—C4—O1	179.3 (3)	C16B—C15B—C20B—C19B	2 (4)
C2—C3—C4—C5	−1.6 (4)	C16B—C17B—C18B—O4B	−179 (2)
C3—C4—C5—N1	176.5 (3)	C16B—C17B—C18B—C19B	−1 (3)
C3—C4—C5—C6	1.5 (4)	C17A—C18A—C19A—N2A	177.5 (3)
C4—C5—C6—C1	−0.2 (4)	C17A—C18A—C19A—C20A	−0.1 (5)
C5—N1—C7—C8	173.2 (3)	C17B—C18B—C19B—N2B	−178.7 (18)
C6—C1—C2—C3	1.1 (4)	C17B—C18B—C19B—C20B	−1 (3)
C7—N1—C5—C4	152.0 (3)	C18A—C19A—C20A—C15A	0.6 (5)
C7—N1—C5—C6	−33.4 (4)	C18B—C19B—C20B—C15B	1 (3)
C7—C8—C9—C10	176.4 (3)	C19A—N2A—C21A—C22	177.3 (3)
C7—C8—C13—C12	−177.5 (3)	C19B—N2B—C21B—C22	−177.2 (15)
C8—C9—C10—O2	−178.0 (3)	C20A—C15A—C16A—C17A	−0.3 (6)
C8—C9—C10—C11	1.4 (4)	C20B—C15B—C16B—C17B	−4 (4)
C9—C8—C13—C12	−1.1 (4)	C21A—N2A—C19A—C18A	−178.0 (3)
C9—C10—C11—O3	178.8 (3)	C21A—N2A—C19A—C20A	−0.5 (6)
C9—C10—C11—C12	−1.4 (4)	C21A—C22—C23—C24	179.7 (3)
C10—C11—C12—C13	0.3 (4)	C21A—C22—C27—C26	−179.8 (3)
C11—C12—C13—C8	1.0 (4)	C21B—N2B—C19B—C18B	175 (2)
C13—C8—C9—C10	−0.1 (4)	C21B—N2B—C19B—C20B	−2 (3)
C14—O2—C10—C9	13.4 (4)	C21B—C22—C23—C24	−176.8 (11)
C14—O2—C10—C11	−166.0 (3)	C21B—C22—C27—C26	174.2 (17)
Cl2A—C15A—C16A—C17A	179.5 (3)	C22—C23—C24—O5	179.5 (3)
Cl2A—C15A—C20A—C19A	179.7 (3)	C22—C23—C24—C25	−0.1 (4)
Cl2B—C15B—C16B—C17B	174.7 (19)	C23—C22—C27—C26	0.7 (4)
Cl2B—C15B—C20B—C19B	−176.9 (18)	C23—C24—C25—O6	−179.7 (3)
O4A—C18A—C19A—N2A	−2.3 (5)	C23—C24—C25—C26	1.2 (4)
O4A—C18A—C19A—C20A	−179.9 (3)	C24—C25—C26—C27	−1.3 (4)
O4B—C18B—C19B—N2B	0 (3)	C25—C26—C27—C22	0.4 (4)
O4B—C18B—C19B—C20B	177 (2)	C27—C22—C23—C24	−0.8 (4)
O5—C24—C25—O6	0.7 (4)	C28—O5—C24—C23	4.9 (5)
O5—C24—C25—C26	−178.5 (3)	C28—O5—C24—C25	−175.5 (3)

### (E)-4-Chloro-2-[(4-hydroxy-3-methoxybenzylidene)amino]phenol Hydrogen-bond geometry (Å, º)

*Cg*2 is the centroid of the C22–C27 ring.

**Table:**

*D*—H···*A*	*D*—H	H···*A*	*D*···*A*	*D*—H···*A*
O1—H1···N1	0.84	2.21	2.695 (3)	116
O3—H3···O2	0.84	2.25	2.690 (3)	113
O4*A*—H4*A*···N2*A*	0.84	2.20	2.675 (4)	116
O6—H6···O5	0.84	2.18	2.645 (3)	115
O1—H1···O3^i^	0.84	2.26	2.854 (3)	128
O3—H3···O6^ii^	0.84	2.02	2.714 (3)	140
O6—H6···O1^iii^	0.84	2.05	2.716 (3)	136
C2—H2···Cl2*B*^iv^	0.95	2.71	3.449 (8)	135
C14—H14*B*···O4*B*^v^	0.98	2.31	2.928 (16)	120
C28—H28*C*···O4*A*^vi^	0.98	2.57	3.142 (4)	117
C7—H7···*Cg*2^i^	0.95	2.90	3.394 (3)	113

Symmetry codes: (i) −*x*, *y*−1/2, −*z*+1; (ii) *x*+1, *y*, *z*; (iii) −*x*−1, *y*+1/2, −*z*+1; (iv) *x*−1, *y*, *z*−1; (v) −*x*+1, *y*+1/2, −*z*+1; (vi) −*x*, *y*+1/2, −*z*+2.
